# Effect of Breed and Finishing Diet on Growth Parameters and Carcass Quality Characteristics of Navarre Autochthonous Foals

**DOI:** 10.3390/ani11020488

**Published:** 2021-02-12

**Authors:** Aurora Cittadini, María V. Sarriés, Rubén Domínguez, Gregorio Indurain, José M. Lorenzo

**Affiliations:** 1 Campus de Arrosadía, IS-FOOD, Institute for Innovation & Sustainable Development in Food Chain, Universidad Pública de Navarra, 31006 Pamplona, Spain; aurora.cittadini@unavarra.es (A.C.); vsarries@unavarra.es (M.V.S.); gindurain@unavarra.es (G.I.); 2 Centro Tecnológico de la Carne de Galicia, rúa Galicia nº 4, Parque Tecnológico de Galicia, San Cibrao das Viñas, 32900 Ourense, Spain; rubendominguez@ceteca.net; 3 Área de Tecnología de los Alimentos, Facultad de Ciencias de Ourense, Universidad de Vigo, 32004 Ourense, Spain

**Keywords:** horsemeat, organic diet, silage, commercial feed, carcass quality, carcass morphology

## Abstract

**Simple Summary:**

Nowadays, populations are more health conscious and pay more attention to the environmental impact of their food choices. In this context, horsemeat plays a significant role, since it is claimed as “dietetic” meat by researchers. In addition, equine meat production is arousing interest due to its relevant environmental potential. However, several factors have been shown to affect carcass and horsemeat quality. In this sense, our study evaluates the impact of breed (Jaca Navarra and Burguete) and type of finishing diet (conventional diet and silage with organic feed) on carcass traits from Navarre endangered foals. Moreover, considering the limited information about Jaca Navarra foals from a meat production perspective, this work represents a starting point to improve the handling of these equids. Data reported Burguete predominance in growth and in most carcass traits, confirming its remarkable aptitude as a meat producer. However, finishing diet showed to improve Jaca Navarra features. A conventional diet showed a positive effect on the majority of variables studied in comparison with the other diet. Thus, breed and the type of finishing diet demonstrated a crucial role in the improvement and optimization of the majority of productive and carcass traits of these endangered equids.

**Abstract:**

This research was conducted to study the effect of breed, Jaca Navarra (JN) vs. Burguete (BU), and finishing diet, conventional concentrate—diet 1 vs. silage and organic feed diet 2—on growth parameters and carcass characteristics from forty-six foals. Parameters as live weight (LW), average daily gain (ADG), body condition score (BCS), and fat depots were monitoring. In addition, the carcass parameters assessed were: carcass weight (CW), conformation, degree of fatness, morphology, and pH. Moreover, hindquarters of the left-half carcasses were sectioned in the main commercial primal cuts of leg. Results showed a clear “breed effect” in favor of BU foals, recording the highest productive values and carcass traits compared with JN foals. On the other hand, finishing diet contributed to improving the productive and carcass features of JN foals. In particular, diet 1 showed to affect positively the features analyzed compared with diet 2. Nevertheless, the meat primal cuts resulted in being unaffected by the breed and diet effects (except for knuckle), obtaining similar values among the groups of animals.

## 1. Introduction

Meat production represents an important sector in the world and, in the European Union (EU), and it is continuously growing [1]. However, in recent years, consumer’s consciousness and food choices have changed, demanding meat not only with a healthy nutritional profile but also environmentally friendly. In this context, horsemeat could easily satisfy these market requirements. Equines, as large framed non-ruminant domestic grazers and hindgut fermenters, can compete advantageously with ruminants for the utilization of pastures and rangelands. In particular, their unique digestive physiology allows an efficient absorption of the n-3 PUFAs from pasture into meat with very low deposition of trans-FA and low methane emissions per unit of meat produced in comparison to ruminants [2,3,4].

Furthermore, it has been pointed out that horses produce meat of an excellent quality and with high nutritional value, characterized by elevated proportion of proteins, iron and vitamin of B group as well as low fat and cholesterol content and a favorable dietetic fatty acids profile [4,5,6,7,8,9,10]. Horses also show high suitability for meat production, presenting higher dressing percentages, closer to 60–70% in meat yield [5,11,12,13,14].

The consumption of horse meat and of its products are still unpopular in many countries, and its production is actually inferior to that recorded for other species such as pork, poultry, bovines, and ovine [3]. On the other hand, owing to its availability and well-known nutritional value, its consumption is slowly increasing as well as the production, registering an average of 700,000 tons per year [2]. In the same manner, in Spain, horse meat consumption is acquiring an important role [11,15]. According to data from the Ministry of Agriculture, Fisheries and Food [16], in Spain, 38,200 equids were slaughtered for meat production in 2019 and the horse meat production increased from 6366 tons in 2009 to 9823 tons, representing an increase of 54.30%. Spain is one of the largest horsemeat producers in Europe, although most of the meat is exported to countries such as Italy or France. Overall, this sector is arousing interest due to its huge social, economic, and environmental potential: keeping the ecosystem of pasturages, protecting the area against fire and erosion, maintaining population in rural areas, reduction of methane and other greenhouse gases, supply food with enhanced n-3 fatty acid content, and preservation of local breeds [2,3,13,17].

The “Jaca Navarra” (JN) and “Burguete” (BU) are autochthonous breeds located in the north of Navarre (NE Spain). These local breeds have been classified as endangered and included in the list of Domestic Animal Diversity Information System hosted by FAO [18]. Burguete is a medium-sized horse breed (6604 animals censed [19]) originated by the crossing of the local mares (Jaca Navarra) and foreign heavy stallion stock (most of them from France such as Trait, Postier, Breton, Percherón, and, more recently, Ardanes and Contois) [20]. Previous studies were conducted about this breed [11,12,21,22,23], defining Burguete as a breed with remarkable potential and aptitude for meat production. On the other hand, Jaca Navarra (1629 animals registered [19]) is a light draught breed whose specific origin is unknown and limited information is available about its characteristics as meat producers [20]. However, it has been established that carcass and horsemeat quality can be influenced not only by breed [11,24], but also by finishing feeding [10,23,25] among others. Generally, foals, after weaning, continue in the pastures until slaughter. Previous to sacrifice, they usually follow a fattening period based on concentrates [14,26,27,28,29,30,31] in order to favor a complete growth and development of the animals and to improve their commercial value. Actually, the production system and feeding strategies applied play a key role in the quality of the horse carcass, fresh meat, or of meat products [6].

In this context, the aim of this study was to assess the effect of breed (Jaca Navarra vs. Burguete) and finishing diet (diet 1 vs. diet 2) on growth parameters, body condition features, and carcass characteristics from forty-six foals.

## 2. Materials and Methods

### 2.1. Experimental Design and Animal Management

For this study, forty-six foals, twenty-seven from Jaca Navarra (JN) and nineteen from Burguete breed (BU), were used. Animals were obtained from local farms after weaning and reared at pasture (for about 5 months) on valley and mountain fields until 17 months of age. Then animals were fattened indoors in the experimental farm of the Institute for Agri-food and Technology and Infrastructures of Navarre (Roncesvalles, Navarre, Spain) with different finishing diets for 3–4 months. Animals were submitted to two dietary regimes identified as diet 1 (D1) and diet 2 (D2). In the D1 group, 22 foals (13 of JN and 9 of BU breeds) were supplemented with conventional concentrates (starter and finisher ones) and straw. The chemical composition of feeds employed, shown in Table 1, was determined according to the standard official methods of AOAC [32]. The starter feed was composed of wheat middling, oats, barley, corn gluten meal, alfalfa, palm kernel, molasses, tallow lard, calcium carbonate, and sodium chloride. The finisher one consists of wheat middling, oats, sunflower flour, molasses, calcium fat, palm oil, calcium carbonate, and sodium chloride. This ration (finisher feed) was complemented with the next mineral/vitamin mix: vitamin A (4800 UI/kg), vitamin D3 (960 UI/kg) and vitamin E (12 mg/kg), mineral expressed in mg/kg manganese (30), zinc (30), magnesium (20), copper (8), iron (8), iodine (0.8), selenium (0.16), and cobalt (0.12).

On the other hand, in D2 group, 24 foals (14 of JN and 10 of BU breeds) were supplemented with silage (produced by local farmers) and an organic fodder (with certification of UE/no UE Organic Agriculture), where silage formed the major part of the diet. Table 1 shows the composition of silage and of the organic concentrate. However, the organic feed was composed of organic oats, organic barley, organic corn, organic wheat, soy oil, calcium carbonate, sodium chloride, and dicalcium phosphate. This ration was complemented with the next mineral/vitamin mix: vitamin A (10,000 UI/kg), vitamin D3 (2000 UI/kg) and vitamin E (15 mg/kg), mineral expressed in mg/kg manganese (65), zinc (50), iron (20), and copper (2).

Each group of foals was gradually introduced to the commercial feeds using oats and silage over a 14-day period before fattening in order to avoid colics that normally appear with a sudden change in the diet. Therefore, animals were separated into four groups: (JN-D1), (JN-D2), (BU-D1), and (BU-D2). Once the fattening period was finalized, foals were transported to an accredited abattoir (Protectora de carne S.L., Salinas de Pamplona, Navarre, Spain) the day before slaughter in compliance with the current EU regulations (Council Regulation 1/2005EC, 2005) [33], without mixing groups and trying to minimize the stress of the animals. All animals were slaughtered at a mean of 21 months. The animals were stunned with a captive bolt, slaughtered and dressed according to the current EU regulations (Council Regulation 1099/2009, 2009) [34].

### 2.2. Growth Parameters and Body Condition Assessment

Bodyweight and body condition evaluation are significant tools for monitoring the health status of an individual horse [35]. Therefore, live weight (LW) of foals in all treatment groups was recorded monthly during the 9 months of the experimental period (from the middle of May 2019 to the middle of February 2020). In addition, the initial weight at the beginning of the finishing feeding period (IW) and live weight at slaughtering (LWS) was obtained for each animal. Average daily weight gain (ADG) in the different phases was calculated. The weighing of animals was normally carried out at the same time of the day with respect to feeding and exercise with the aim of minimizing the influence of normal daily variability on measurements according to Carter et al. [35].

Body condition evaluation was measured by two methods: body condition score (BCS) and ultrasonic measurement of subcutaneous fat depth. All measurements were realized the day before slaughter. In particular, the BCS system was used to monitor adiposity of foals. This method is based on visual and physical evaluation of subcutaneous fat deposition in specific body regions. It consists of a rating of body condition based on a numerical scale with specific criteria for each category. For this study, the 0–5-point scale described by Carroll et al. [36] was used and determined by two trained evaluators. This BCS system evaluates subcutaneous fat deposition in 4 body areas (neck, back, ribs, and pelvis) on a scale of 0 (very poor) to 5 (very fat) [35].

Nevertheless, scorer experience, horse breed, gender, or local fat deposits could be confounding factors. Therefore, ultrasonography assessment of subcutaneous fat thickness (SFT) considered a more accurate, objective, and non-invasive method, was employed to measure fat distribution in live horses [35,37]. The determination of SFT was carried out at the level of seven anatomic locations (over 25%, 50%, and 75% of neck length, behind the shoulder, over the ribs, over the rump, and over the tailhead) as described by Martin-Gimenez [37]. All measurements of SFT were performed via B-mode with commercial ultrasonographic imaging system (Sonovet 600 Ultrasound, Medison Co. Ltd., Seoul, Korea) equipped with a 3.5 MHz linear transducer (120 × 20 mm). All images were taken on the left side while the equids were standing in a normal position and individually held back to minimize movements. Sunflower oil was used as coupling medium. The scanning and the interpretation of the images of each zone were performed in triplicate by the same researcher to avoid variability in the measurement technique. Moreover, the probe was positioned perpendicular to the floor. Anatomic landmarks used to guide the transducer placement in the neck area were located at the interface between the crest and neck musculature, identified by palpation and visual assessment at: 25% (SFT-N25%), 50% (SFT-N50%), and 75% (SFT-N75%) of neck length [38]. As regards the measurements on the trunk, the probe was situated as previously described [39,40] at: the area just caudal to the shoulder (SFT-S), the area between 12th to 13th ribs (SFTR12-13), the rump (SFT-R), and the tailhead (SFT-TH).

### 2.3. Carcass Measurements

Immediately after slaughter, hot carcass weight (HCW) was determined, and dressing percentage (DP) was calculated [12]:

(1) $$DP=\frac{HCW}{LWS}\;\times 100$$

Subsequently, the carcasses were chilled for 24 h in a conventional room with a temperature of 0 °C and cold carcass weight (CCW) was determined.

Carcasses were classified at 24 h post-mortem by qualified slaughterhouse personnel. Conformation and degree of fatness are potential tools for classifying and predicting foal carcass quality. However, considering the limitations of the ‘Catalogue de Classement des Équidés’ carcass classification from the ONIBEV (1979) described by Fabregas et al. [41], an alternative method was followed: a body conformation (EUROP) (for foals over 12 months of age) and fat cover classification described by Italian researchers [42], who considered the development of the carcass profiles, with particular attention to the leg, loin and back. This method could be considered an outspread adaptation of the Community model for the classification of heavy beef carcasses [43], normally employed in the equine sector. The EUROP conformation system consists of a 15-point scale ranging from 1 (very bad conformation) to 15 (very good conformation): P−, P, P+, O−, O, O+, R−, R, R+, U−, U, U+, E−, E, E+. On the other hand, fatness score, based on the amount and distribution of the fat in the external and internal parts of the carcass, includes a scale from 1 (very low fat) up to 15 (very high fat): 1−, 1, 1+, 2−, 2, 2+, 3−, 3, 3+, 4−, 4, 4+, 5−, 5, 5+.

Image capture and video image analysis (VIA) technology measurements were used in order to assess carcass morphology. Actually, the day after the slaughter, the left side of the carcass was immobilized carefully before the process of capturing images from the dorsal, lateral, and medial views, using a high-resolution digital camera (Olympus, E300, Tokyo, Japan). The camera was set as follows: manual operation mode, F/4.2, ISO velocity 200, flash off, focal distance 24 mm. The captured images were saved as 2448 × 3264 pixel JPEG format. All images were captured with the same light and camera conditions. The camera was placed at 4 m from the carcass side in a fixed position perpendicular to the carcass and at a height of 1.70 m from the floor. As a scale, a 15 cm plastic ruler was used. From those images, video image analysis software (ImageJ, 1.52t, U.S. National Institutes of Health, Bethesda, MD, USA;, http://imagej.nih.gov/ij/) was employed to obtain the following morphometric measures: carcass length (CL), width of leg (WL), external depth of chest (EDC), compactness index (CCI), perimeter (CP), and area (CA) [44]. The information published in several articles was used to set the VIA carcass measurements [5,13,45,46,47,48]. Moreover, fat thickness (FT) was measured at the level of the 9th rib with a stainless steel ruler [49]. The compactness index (CI) was calculated as cold carcass weight divided by carcass length [12].

Additionally, pH was measured at the level of the 6th rib of the left carcass with a portable pHmeter (Crison 507, Hach Lange Spain, Barcelona, Spain) with a penetrating electrode and temperature measurement [50]. The values of pH were obtained 45 min later carcass dressing (pH_45 min_) and again 24 h post-mortem (pH_24 h_). Finally, once in the cutting room (Cárnicas Mutiloa, Rocaforte, Navarre, Spain), the left-half carcass was divided in order to obtain the main leg cuts (%): topside (*semimembranous, glacilis, adductor and pectineus*), rump (*gluteus medius, iliacus, gluteus accessorious, gluteus profundus*), silverside with eye round (*biceps femoris and semitendinosus*) and knuckle (*vastus intermedius, vastus lateralis, vastus medialis, rectus femoris and tensor fasciae latae*). These cuts contribute to the determination of final economic value of a horse carcass. They also have a relevant commercial value both for consumption and meat products’ elaboration. 

### 2.4. Statistical Analysis

An ANOVA using the General Lineal Model (GLM) procedure of the SPSS package (SPSS 25.0, Chicago, IL, USA) was performed for all variables considered in the study. Fixed effect of breed (B) and finishing diet (FD) were included in the model. The model used was:

Y_ij_ =μ + B_i_ + FD_j_ + (B × FD)_ij_ + ε_ij_ (2)

where Y_ij_ is the observation of dependent variables, μ the overall mean, B_i_ the effect of breed, FD_j_ the effect of finishing diet, B×FD is the effect of interaction of the _i_th breed and the _j_th finishing diet, and ε_ij_ is the residual random error associated with the observation. Interaction B×FD was included in the model, only when significance was showed. IW was introduced as covariate. In all analyses, least squares mean were compared using Duncan’s *t*-test. Significance was declared at *p* ≤ 0.05.

## 3. Results and Discussion

### 3.1. Effect of Breed and Finishing Diet on Productive Variables

The growth parameters of horses are shown in Table 2. Data indicated that the growth parameters were affected by breed. These results are in agreement with those reported by other authors [11,24,26], who highlighted that growth indices can be affected by the foal breed. During the fattening period, breed influenced significantly the final growths (*p* < 0.001), where Burguete foals generated the highest weights and ADG values. However, the highest difference takes place at slaughtering, with the BU foals being 124.78 kg heavier than the JN ones. This trend is consistent with data reported by other authors [21], who observed a clear “breed effect” in favor of Burguete foals in growth parameters compared with Jaca Navarra foals fattened with the same diet. These results could be expected considering the aforementioned different breed size and its IW values.

Weights at slaughtering for BU group were highly greater than those found by other authors [11,12], in Burguete foals slaughtered at 24 months of age (395 kg). On the other hand, Villanueva et al. [21], studying the effect of finishing diet in Burguete foals, reported results for 17-month-old animals similar to ours (564 kg). In the same way, Burguete foals presented weights consistent with those reported on adult Malopolska and Silesian Horses [15] and with 18-month-old Italian Heavy draft horses reported by other authors [28]. As regards Jaca Navarra foals LWS, results were slightly greater than those obtained by animals of the same breed (400.7 kg) [21]. On the other hand, similar outcomes were observed in 16-month-old Burguete foals (mean value of 411.3 kg) [12] and 18-month-old Sanfratellano foals (411.00 kg) [24].

Regarding ADG, BU recorded significantly higher values (1.14 kg/day) compared with the JN group (0.74 kg/day) (*p* < 0.001). However, our ADG values in the finishing period are slightly lower than those reached by animals (17 months old) of the same breeds on intensive systems [21], probably due to the different age and duration of fattening period (3–4 months vs. 2–3 months, respectively). On the other hand, ADG values for JN foals were similar to those reported by other authors, studying cross breeding foals (Galician Mountain x Hispano Bretón) (GM X HB) [25,26].

In addition, the growth of foals was highly affected by the type of diet. Regarding LWS, between D1 and D2 groups, it was different (*p* < 0.001). ADG at slaughter also showed relevant difference (*p* < 0.001), where D1 foals presented highly greater ADG values than another group (1.04 kg/day vs. 0.78 kg/day, respectively). These outcomes are consistent with was previously reported by other authors [14,26,27], who confirmed that the livestock production system and amount of concentrate may affect the growth of foals. Furthermore, these results may be expected, considering the different ratio of diets and its composition, D2 is mainly composed by silage complemented with an organic feed and the conventional concentrates showed a greater fat percentage compared with the “ingredients” of D2. Considering the four groups, it is evident that BU-D1 had the highest values, followed by the BU-D2 group. The BU-D1 group duplicates the ADG values of the JN-D2 one. Moreover, results showed that diet caused significant differences in LWS mainly within BU groups compared to JN ones. On the other hand, any significant interactions between the main categories (breed x finishing diet) were found (*p* > 0.05).

Carcass features are shown in Table 2. CW as well as DP and CCI were significantly affected (*p* < 0.05) by breed. On the other hand, finishing diet affected CW, DP, CL, WL, CA, CCI, and fat thickness (*p* < 0.05). LW (as commented above) and HCW were significantly (*p* < 0.001) different between breeds and type of diets, with the lowest values for JN foals and the highest for the BU and in foals belonging to D1 group, reaching a mean value of 349 kg. However, there is a great heterogeneity among European equine carcasses coming from the different horse origin (breed and husbandry system). Consequently, it is difficult to compare results with those of other researches, as foals are of diverse ages and breeds.

Villanueva et al. [21], studying the effect of the fattening diet on 17-month-old foals of the same breeds, obtained CW similar to ours. On the other hand, in our study, BU carcasses were greater than those usually obtained from old animals (24 months) of the same breed [11,12] and from other specialized breeds for meat production as Hispano-Bréton [11] or Sanfratellano [24]. These authors reported CW of 266 and 259 kg, 275 kg and 244 kg, respectively. Considering DP, statistical analysis showed significant differences (*p* < 0.05) among JN and BU foals, as it could be expected. Similarly, the type of finishing diet influenced significantly (*p* < 0.001) foal yields. In fact, animals with a conventional diet recorded the highest values (62.81%) compared with the other group (59.57%) (data not shown). These results are in agreement with data previously reported by other studies [12,21,24], where foal meat gave yields among 59% and 63%.

As regards carcass measurements, data did not show significant differences among the two breeds (*p* > 0.05); however, BU foals recorded the highest values. This fact was expected since BU are bigger animals and is in line with what was discussed regarding live weight and carcass weight. In fact, JN animals showed shorter carcasses than BU ones (209.85 vs. 222.53 cm). In addition, variables related with the leg and chest volume such as WL and EDC were also higher for BU foals. As regards diet, D1 recorded greater values for CL (*p* < 0.001) and WL than the D2 group (*p* < 0.01). Our data for WL and EDC from BU are slightly greater than those found by other authors [44], who observed in Burguete foals of 16–18 months-old values of 41.4 cm and 55.1 cm, respectively, that are closer to the values obtained from JN foals. Considering CL, our data for JN and BU foals seem to be consistent and greater, respectively, with results reported by previously studies [5,51].

CCI indicates the carcass compactness, and it was significantly (*p* < 0.001) greater in BU than in JN foals (1.45 vs. 1.18) and in the D1 group than in the D2 group (1.24 vs. 1.34). Moreover, results showed that the JN group had a smaller carcass perimeter and area. However, although these differences were not statistically significant, these values remained in line with the morphological data obtained in this study. In the same way, breed did not affect FT values (*p* > 0.05). Nevertheless, BU foals presented slightly greater values than JN ones (a mean of 3.79 vs. 3.58 mm, respectively). On the other hand, statistical analysis showed that the type of finishing diet affected (*p* < 0.05) fat thickness. There were significant (*p* < 0.001) differences among the two diet groups, where the D2 group presented less fat values compared with the D1 group (a mean of 3.40 vs. 3.95 mm, respectively). It is worth noting that the biggest differences between the two types of diet were recorded among Burguete foals, where BU-D1 recorded the highest values (4.32 mm). On the other hand, our values did not agree with those recorded by Ruiz et al. [25], who observed lesser fat thickness compared to ours in crossing breed foals (GM × BU) fattened with standard finishing feed (3.18 mm). However, other authors [12] showed much greater values compared with us, recording a mean of 7 mm in 24-month-old Burguete foals and a mean of 5.71 mm in 16-month-old Burguete foals. On the whole, carcass characteristics were unaffected (*p* > 0.05) by the interaction of breed and type of diet except for the values of fat thickness and pH. However, pH_45 min_ values were consistent with outcomes found by other authors [12]. Conversely, data obtained at 24 h post-mortem resulted in being slightly higher than those normally obtained in foal meat, ranging from 5.57 to 5.61 [11,12].

**Figure 1:**
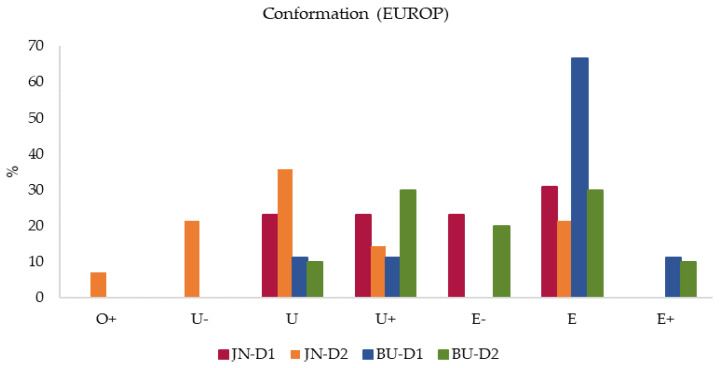
Percentage distribution of conformation of Jaca Navarra (JN) and Burguete (BU) foals fattened with two different finishing diets: D1 (Diet 1) = conventional concentrate, D2 (Diet 2) = Silage + organic concentrate.

**Figure 2:**
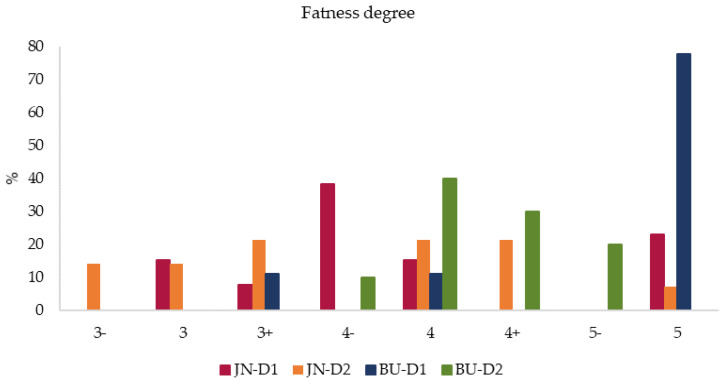
Percentage distribution of fatness degree of Jaca Navarra (JN) and Burguete (BU) foals fattened with two different finishing diets: D1 (Diet 1) = conventional concentrate, D2 (Diet 2) = Silage + organic concentrate.

Figure 1 and Figure 2 show the percentage distribution of the carcasses in each category of conformation and degree of fatness according to the four groups in study. Graphics show that there were differences in the distribution of the foal carcasses among the various conformation and degree of fatness classes. In particular, 67% of BU-D1, 31% of JN-D1, and 30% of BU-D2 foals were scored in category E, whereas 36% of JN-D2 were classified in category U. Concurrently, 78% of BU-D1 were over-finished and included in category 5 (very high fat cover), 40% of BU-D2 foals were scored in category 4 (abundant fat cover) and 38% of JN-D1 group was classified in the slightly minor class 4-. On the other hand, 21% of JN-D2 foals were equally scored in categories 3+, 4, and 4+. Thus, looking at the groups, BU-D1 foals obtained the highest scores both for conformation and adiposity degree, while JN-D2 ones recorded the lowest values. These results agreed with the aforementioned results about productive variables, where BU-D1 foals reported the highest LWS, carcass measurements, and FT values among the four groups. On the whole, all groups obtained good conformation and adiposity scores with a good muscular development and abundant fat cover. Again, our values cannot be easily compared with data obtained from other studies; in this case, mainly because a broader conformation scale [42] was used compared with previous studies. In addition, different values were obtained by Sarriés et al. [12], who found smaller values in Burguete foals both for conformation and fat degree, corresponding in our 15-point conformation scale to R and 3 categories, respectively. Similarly, our conformation values for BU foals were slightly higher than data obteined by Ruiz et al. [25], who reported for GM x BU equids values corresponding in the EUROP scale to E^−^. On the other hand, the BU-D1 group recorded fatness values according to the values obtained by the same authors [25] from 26-month-old foals, coinciding with category 5.

### 3.2. Effect of Breed and Finishing Diet on Main Commercial Meat Cuts

Table 3 shows the data obtained by the dissection of the hind quarter (HQ) of the half carcass in the commercial primal cuts. In particular, data were similar independently by breed (*p* > 0.05), except for the knuckle, where BU reported higher values (*p* < 0.01). Although JN foals recorded slower percentages than BU, values of the different cuts (topside, and silverside and eye round) did not vary in a significant manner (a mean of 5.34 vs. 5.37% and 7.80 vs. 7.88%, respectively). In the same way, the type of diet did not affect the results significantly (*p* > 0.05), except for the knuckle value. In fact, the knuckle of D2 group occupied a greater percentage (*p* < 0.05) than the D1 group (a mean of 4.08 vs. 3.91%, respectively). Furthermore, any significant interactions among the main categories (breed x finishing diet) were observed (*p* > 0.05).

Considering the singular cuts, other authors [26] found in 15-month-old GM and GM x HB foals, finished with 1.5 kg of concentrate, topside values ranging between 5.33 and 5.42% as observed in this study. Similarly, our values for knuckle (among 3.90 and 4.13%) are in agreement with those previously reported [13,25,27,45], in Galician Mountain (GM) and GM × BU foals of different ages and livestock systems. Values obtained by silverside and eye round were approximately similar to those obtained in another research work [25], studying GM × BU finished with conventional feed. Nevertheless, values for rump, in a range of 6.35 and 6.69%, occupied a greater percentage compared with those obtained in previous studies [25,26,27], in GM and cross breeding foals finished with concentrate. Overall, it is complex comparing our values, not only for the limit number of studies about JN and BU foals, but also because, as aforementioned, in previous studies, different breeds, ages, sex, and livestock system were used and animals, in some cases, were reared in different periods of the year. In addition, examining primal cuts’ values, differences may also appear due to the use of different dissection procedures. For instance, previous studies used the methodology described by Carballo et al. [52]. However, our data did not reflect the significant differences detected in the conformation variables and measurements as BCS, DP, and CCI. These productive variables, usually used to evaluate the muscular development of carcasses, seem to not agree with the commercial evaluation of carcasses, determined by the proportion of the main commercial cuts.

### 3.3. Effect of Breed and Finishing Diet on the Subcutaneous Fat Thickness and Body Condition Scores Measurements in Different Anatomic Regions

Considering ultrasonographic analysis, data showed (Table 4) that breed affected the subcutaneous fatness. Burguete presented the greater values compared to Jaca Navarra foals; in particular, significant differences were obtained at the level of neck, SFT-N25%, and SFT-N50%, of the ribs and of the shoulder (*p* < 0.05). These results are consistent with data reported by other authors, which confirmed that breed could affect these fat depots [6].

Considering the type of diet, animals fattened with D1 reported the highest values, in particular at the level of the neck (SFT-N75%) and of the rump (SFT-R). Although, in this work, subcutaneous fat values obtained were much more minor than those obtained in 2–5-year-old Andalusian horse by other authors [37]. In general, as expected, the data obtained from the ultrasonography analysis showed the same trend observed by the carcass measurements and characteristics and degree of fatness exposed through the present manuscript. As regards BCS, statistical analysis showed that there were significant differences among the two breeds (*p* < 0.05), while diet did not affect this variable (*p* < 0.05). In fact, the four foal groups presented similar scores close to 5, representing the condition between fat and obese animals [36]. Thus, data confirmed that the application of these methods in living animals, in particular, ultrasonic measurement of subcutaneous fat depth, could represent a reference method to detect and predict the carcass fatness level and to avoid the excessive adiposity at the moment of slaughtering (as occurred in BU-D1 foals). Actually, an excessive adiposity could penalize the commercial value of the carcass in the market. 

## 4. Conclusions

From the results, we can conclude that breed and the type of finishing diet had a significant influence on growth parameters and on most of the carcass traits. The main differences between JN and BU foals were found in LW, ADG, CW, DP and in some morphological measurements confirming the notable aptitude for meat production of the Burguete breed. Assuming from the beginning that the two breeds are characterized by different size, morphometric measures recorded a similar tendency. On the other hand, finishing diet contributed to improving the productive and carcass features of JN foals. In particular, D1 showed to positively affect the features analyzed compared with D2. Conformation and fatness scores confirmed the predominance of Burguete foals, especially the BU-D1 group. However, it is worth noting how D1 improved JN conformation. However, the meat primal cuts resulted in being unaffected by the breed and diet effects (except for knuckle), obtaining similar values among the groups of animals. These results can be considered positively by a commercial point of view.

In addition, future investigations could be planned about the application of ultrasonic fat measurements along the fattening period and the determination of the variables that could better predict the carcass yields. Finally, further studies are necessary to establish a production system able to valorize these autochthonous foal breeds and to improve the quality standards of them, from the carcass to the final meat product. Otherwise, considering the limited information about the quality of Jaca Navarra breed as meat producers, this study could represent a starting point to characterize and to improve the handling of these endangered equids.

## Figures and Tables

**Table 1:** Chemical composition (expressed as percentage) of oats, concentrates, and silage allocated to the foals during the fattening period.

	Oats	D1		D2	
		Starter Feed	Finisher Feed	Silage	Organic Feed
Moisture	11.64	-	-	-	-
Ash	2.59	8.5	4.5	8.81	5.00
Protein	8.78	12.9	12.8	16.22	8.50
Crude fiber	12.90	14.00	10.40	-	7.00
Crude fat	5.58	3.4	6.00	-	4.80
Starch	40.00	-	-	-	-
Phosphorus	0.26	-	-	-	-
Calcium	0.08	-	-	-	-
Sodium	-	1.00	0.30	-	0.30
Methionine	-	-	-	-	0.20
Lysine	-	-	-	-	0.30
Dry matter	-	-	-	77.90	-
Acid detergent fiber	-	-	-	26.50	-
Neutral detergent fiber	-	-	-	56.30	-

D1 (Diet 1) = conventional concentrate, D2 (Diet 2) = Silage + organic concentrate.

**Table 2:** Effect of breed and finishing diet on productive variables, carcass measurements, and carcass pH values from Jaca Navarra (JN) and Burguete (BU) foals.

Parameters	JN		BU		SEM	Significance		
	D1	D2	D1	D2		B	FD	B x FD
IW (kg)	333.38 ^a^	322.57 ^a^	407.11 ^b^	405.20 ^b^	8.223	***	ns	ns
LWS (kg)	428.92 ^a^	400.79 ^a^	560.33 ^c^	520.00 ^b^	11.308	***	***	ns
ADG (kg/day)	0.83 ^b^	0.65 ^a^	1.33 ^c^	0.96 ^b^	0.044	***	***	ns
HCW (kg)	270.87 ^b^	238.34 ^a^	349.39 ^d^	310.94 ^c^	7.267	***	***	ns
CCW (kg)	265.45 ^b^	233.57 ^a^	342.40 ^d^	304.72 ^c^	7.122	***	***	ns
DP (%)	63.12 ^b^	59.42 ^a^	62.36 ^b^	59.78 ^a^	0.310	*	***	ns
CL (cm)	215.46 ^b^	204.65 ^a^	227.17 ^c^	218.35 ^b^	1.862	ns	***	ns
WL (cm)	42.60 ^b^	38.94 ^a^	45.02 ^b^	43.12 ^b^	0.521	ns	**	ns
EDC (cm)	58.69 ^a^	56.65 ^a^	62.44 ^b^	62.11 ^b^	0.569	ns	ns	ns
CCI (kg/cm)	1.23 ^b^	1.14 ^a^	1.51 ^d^	1.40 ^c^	0.025	***	***	ns
CP (cm)	594.95 ^a^	582.94 ^a^	631.78 ^b^	626.51 ^b^	4.509	ns	ns	ns
CA (cm^2^)	9509.62 ^b^	8869.78 ^a^	10705.81 ^c^	10428.20 ^c^	148.604	ns	**	ns
FT (mm)	3.70 ^b^	3.47 ^ab^	4.32 ^c^	3.31 ^a^	0.075	ns	***	**
pH_45min_	6.51	6.44	6.42	6.51	0.017	*	ns	*
pH_24h_	6.00 ^b^	5.98 ^ab^	5.90 ^a^	6.04 ^b^	0.015	ns	ns	**

SEM. Standard error of the mean; Sig. Significance; *** (*p* < 0.001), ** (*p* < 0.01), * (*p* < 0.05), ns. (not significant); D1 (Diet 1) = conventional concentrate, D2 (Diet 2) = Silage + organic concentrate; B = Breed; FD = Finishing diet; ^a–d^ Within a row, means without a common superscript are significantly different (*p* < 0.05; Duncan´s test); IW = initial weight at start of finishing diet; LWS = Live weight at slaughtering; ADG = Average daily gain; HCW = Hot carcass weight; CCW = Cold carcass weight; DP = Dressing percentage; CL = Carcass length; WL = Width of leg; EDC = External depth of chest; CCI = Carcass compactness index; CP = Carcass perimeter; CA = Carcass area; FT = Fat thickness.

**Table 3:** Effect of breed and finishing diet on main commercial meat cuts of leg from Jaca Navarra (JN) and Burguete (BU) foals.

	JN		BU		SEM	Significance		
	D1	D2	D1	D2		B	FD	B x FD
Hind quarter %								
Topside	5.34	5.33	5.30	5.44	0.040	ns	ns	ns
Rump	6.56	6.61	6.35	6.69	0.094	ns	ns	ns
Knuckle	3.90 ^a^	4.04 ^ab^	3.93 ^ab^	4.13 ^b^	0.034	**	*	ns
Silverside with eye round	7.97	7.64	7.83	7.93	0.090	ns	ns	ns

SEM. Standard error of the mean; Sig. Significance; * (*p* < 0.05), ns. (not significant); D1 (Diet 1) = conventional concentrate, D2 (Diet 2) = Silage + organic concentrate; B = Breed; FD = Finishing diet; ^a,b^ Within a row, means without a common superscript are significantly different (*p* < 0.05; Duncan´s test). ** (*p* < 0.01).

**Table 4:** Effect of breed and finishing subcutaneous fat thickness (SFT) and body condition scores (BCS) measurements in different anatomic sites from Jaca Navarra (JN) and Burguete (BU) foals.

	JN		BU		SEM	Significance		
	D1	D2	D1	D2		B	FD	B x FD
SFT-N25% (mm)	2.05 ^a^	1.88 ^a^	2.89 ^b^	2.87 ^b^	0.102	***	ns	ns
SFT-N50% (mm)	1.79 ^a^	1.55 ^a^	2.41 ^b^	2.27 ^b^	0.087	*	ns	ns
SFT-N75% (mm)	3.05 ^ab^	2.52 ^a^	3.63 ^b^	3.13 ^b^	0.111	ns	*	ns
SFTR 12–13 (mm)	2.54 ^a^	2.45 ^a^	3.48 ^b^	3.37 ^b^	0.118	*	ns	ns
SFT-R (mm)	1.69 ^ab^	1.45 ^a^	1.96 ^b^	1.33 ^a^	0.066	ns	***	ns
SFT-TH (mm)	1.82	1.40	1.93	1.70	0.099	ns	ns	ns
SFT-S (mm)	1.82 ^a^	1.83 ^a^	2.70 ^b^	2.50 ^b^	0.104	*	ns	ns
BCS (mm)	4.70	4.59	4.58	4.57	0.036	*	ns	ns

FD^1^ At start finishing diet; S^2^ at slaughtering; SEM. Standard error of the mean; Sig. Significance; *** (*p* < 0.001), ** (*p* < 0.01), * (*p* < 0.05), ns. (not significant); D1 (Diet 1) = conventional concentrate, D2 (Diet 2) = Silage + organic concentrate; B = Breed; FD = Finishing diet; ^a–b^ Within a row, means without a common superscript are significantly different (*p* < 0.05; Duncan´s test); SFT-N(25%–50%–75%) = Subcutaneous fat thickness of 25%–50%–75% of neck length; SFT-R12-13 = Subcutaneous fat thickness between 12th and 13th ribs; SFT-R = Subcutaneous fat thickness of rump; SFT-TH = Subcutaneous fat thickness of tailhead; SFT-S = Subcutaneous fat thickness of shoulder; BCS = Body condition score.

## Data Availability

Not applicable.
